# Case Report: Eccrine spiradenoma of the shoulder mimicking a vascular lesion on ultrasonography and CT

**DOI:** 10.3389/fmed.2026.1948246

**Published:** 2026-09-15

**Authors:** Qiyao Yu, Jinxin Hu, Bin Xiao

**Affiliations:** Department of Plastic Surgery, BOE Hospital, Chengdu, China

**Keywords:** adnexal tumor, case report, computed tomography, eccrine spiradenoma, histopathology, shoulder, ultrasonography, vascular mimics

## Abstract

Eccrine spiradenoma is an uncommon benign cutaneous adnexal tumor that presents as a painful dermal or subcutaneous nodule. Prominent vascularity may cause the lesion to resemble a vascular anomaly on imaging. We report a 27-year-old man with a painful right shoulder mass of 4 years’ duration. Ultrasonography suggested a vascular malformation with possible thrombosis, while contrast-enhanced CT was interpreted as a vascular lesion. Complete excision was performed, and histopathology revealed the characteristic lobulated architecture, dual cell population, and focal ductal differentiation of eccrine spiradenoma. At 12-month follow-up, there was no clinical evidence of recurrence. This case shows that an adnexal tumor should remain in the differential for painful solid superficial masses despite conspicuous Doppler flow and enhancement. When imaging remains indeterminate, clinical correlation and histopathologic confirmation are important.

## Introduction

Eccrine spiradenoma (ES) is an uncommon benign cutaneous adnexal tumor, originally described by Kersting and Helwig in 1956 (1). It typically presents as a solitary, slow-growing dermal or subcutaneous nodule, with pain or tenderness being frequent features (2).

Because the clinical appearance is non-specific, imaging is often obtained to assess superficial masses. On ultrasonography, ES usually appears as a solid hypoechoic lesion in the deep dermis or subcutaneous fat, with posterior acoustic enhancement and variable internal Doppler flow (3–6). When vascularity is prominent, ES—particularly the giant vascular variant—may be mistaken for a vascular tumor or vascular malformation (7–11). Distinguishing these entities is clinically relevant because a presumed vascular anomaly may lead to additional vascular imaging or intervention, whereas ES ultimately requires histopathologic confirmation and is generally managed by excision. We report a shoulder ES that mimicked a vascular lesion on ultrasonography and CT and discuss the practical points that complicated the preoperative assessment.

## Case description

A 27-year-old man presented with a right shoulder mass that had enlarged slowly over 4 years and remained tender. There was no history of trauma or previous surgery involving the right shoulder and no family history of similar lesions or skin tumors. He was unemployed at presentation and reported no relevant occupational, chemical, or radiation exposure. The complete blood count and coagulation profile were within reference ranges, with a high-sensitivity C-reactive protein level of 1.44 mg/L.

Physical examination revealed a subcutaneous nodule measuring approximately 4 cm × 3 cm on the right shoulder. The lesion was moderately firm with ill-defined margins and limited mobility, and it was tender on palpation. The overlying skin was intact without ulceration or color change, but superficial tortuous vessels were visible and the local skin appeared thinned. A preoperative clinical photograph was not retained, which limits retrospective assessment of the gross cutaneous appearance.

## Diagnostic assessment, therapeutic intervention, and follow-up

Color Doppler ultrasonography performed at Hospital 1 on 12 April 2024 demonstrated a reticular and tubular echoic lesion centered in the subcutaneous layer, measuring approximately 5.0 cm × 3.2 cm × 0.9 cm and closely apposed to the dermis. The margins were indistinct and the shape was irregular. Punctate and linear intralesional flow was observed, with both venous and arterial spectral signals recorded. The study was interpreted as “vascular malformation with possible thrombosis” (Figure 1A).

**Figure 1:**
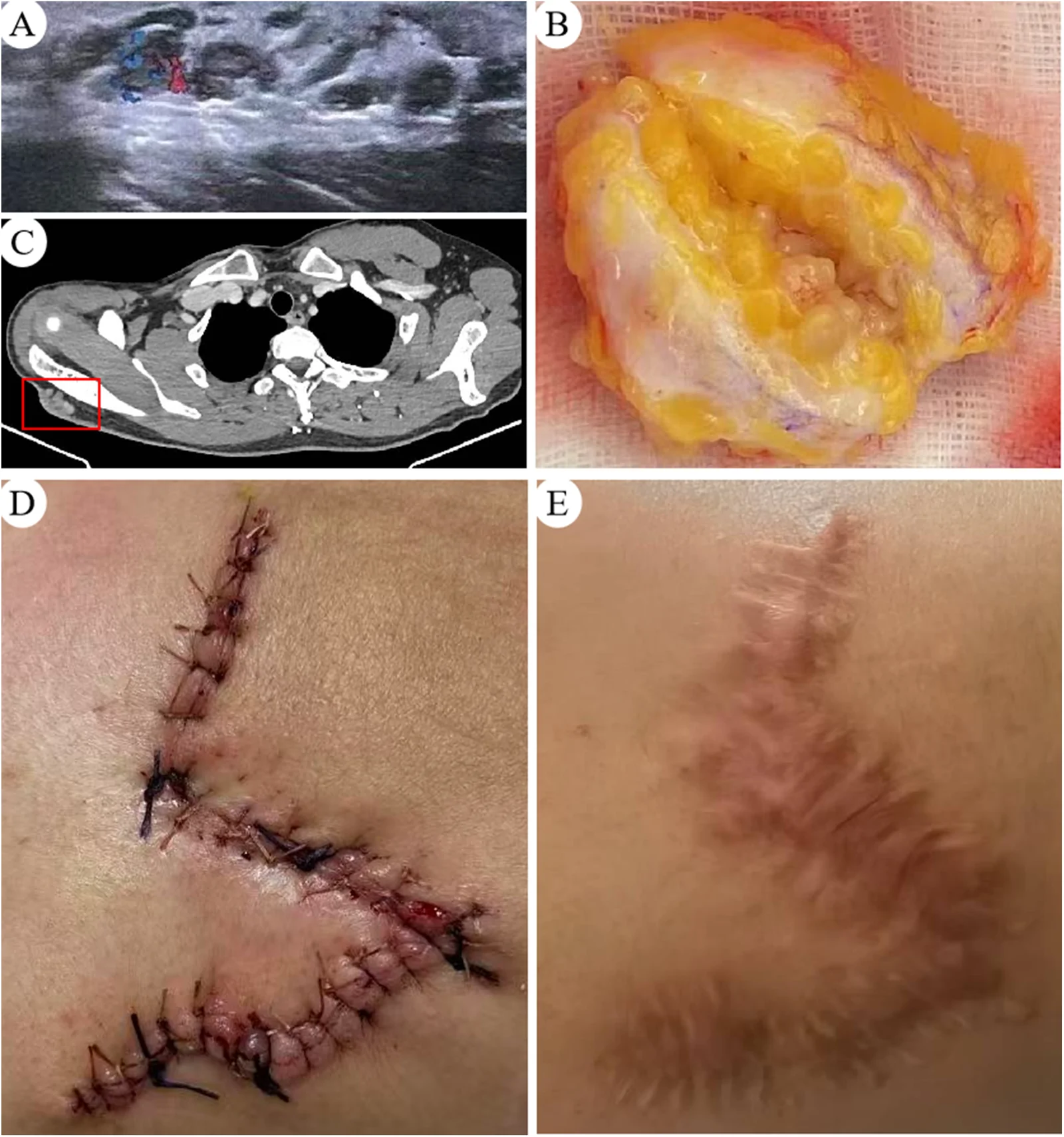
Imaging, gross specimen, and postoperative outcome. **(A)** Preoperative color Doppler ultrasonography showing a reticular and tubular lesion in the right shoulder subcutaneous layer with internal Doppler flow. **(B)** Gross specimen with attached adipose tissue. **(C)** Preoperative axial contrast-enhanced CT showing serpiginous, beaded subcutaneous soft-tissue nodules with mild-to-moderate heterogeneous enhancement in the right scapular region. **(D)** Immediate postoperative appearance. **(E)** Surgical site at 12-month follow-up.

Thin-slice contrast-enhanced CT performed at Hospital 2 on 15 May 2024 showed subcutaneous soft-tissue density nodules in the right scapular region with a serpiginous, beaded distribution over approximately 45 mm × 15 mm × 12 mm. The lesion showed mild-to-moderate heterogeneous enhancement on axial images. No definite osseous abnormality was identified. The original report described the lesion as “subcutaneous plexiform hemangioma” (Figure 1C).

Based on these original clinical and imaging interpretations, the leading considerations were a vascular malformation with thrombosis and a vascular tumor. Given the persistent pain and uncertain diagnosis, surgical excision was planned for both diagnosis and treatment.

On 16 May 2024, the patient underwent complete excision of the right shoulder mass with local flap closure. A 4 cm longitudinal incision was made over the lesion. The intraoperative mass measured approximately 4 cm × 3 cm × 1 cm and extended from the deep dermis into the subcutaneous tissue. The operative record described a clear gross boundary without obvious adhesion or tortuous vessels in the excised field. The lesion was removed *en bloc* together with part of the thinned overlying skin. A rhomboid skin flap measuring approximately 4 cm × 4 cm was designed inferior to the defect, rotated into the wound, and closed with interrupted sutures to reduce tension. Estimated blood loss was about 5 mL. Postoperative care included routine dressing changes, planned suture removal at about 14 days, and temporary limitation of excessive shoulder movement.

The pathology specimen measured 3.7 cm × 2.8 cm × 1.4 cm and was received partially opened. The cut surface was gray-white to gray-yellow, solid, and of moderate consistency; the yellow tissue visible around the specimen in Figure 1B represents attached adipose tissue. No gross hemorrhage was described in the pathology report. Microscopy revealed a lobulated dermal tumor without a complete capsule. Tumor nests contained two cell populations, with small basaloid cells showing hyperchromatic nuclei at the periphery and larger pale epithelial cells centrally. Focal duct-like structures were present, and the stroma contained dilated congested vessels with focal basement membrane-like hyalinized material (Figure 2). The diagnosis of eccrine spiradenoma was rendered by the hospital pathology department and reviewed by the attending pathologist. The characteristic hematoxylin and eosin morphology was considered sufficient for diagnosis. Immunohistochemistry was not performed because the patient declined further testing.

**Figure 2:**
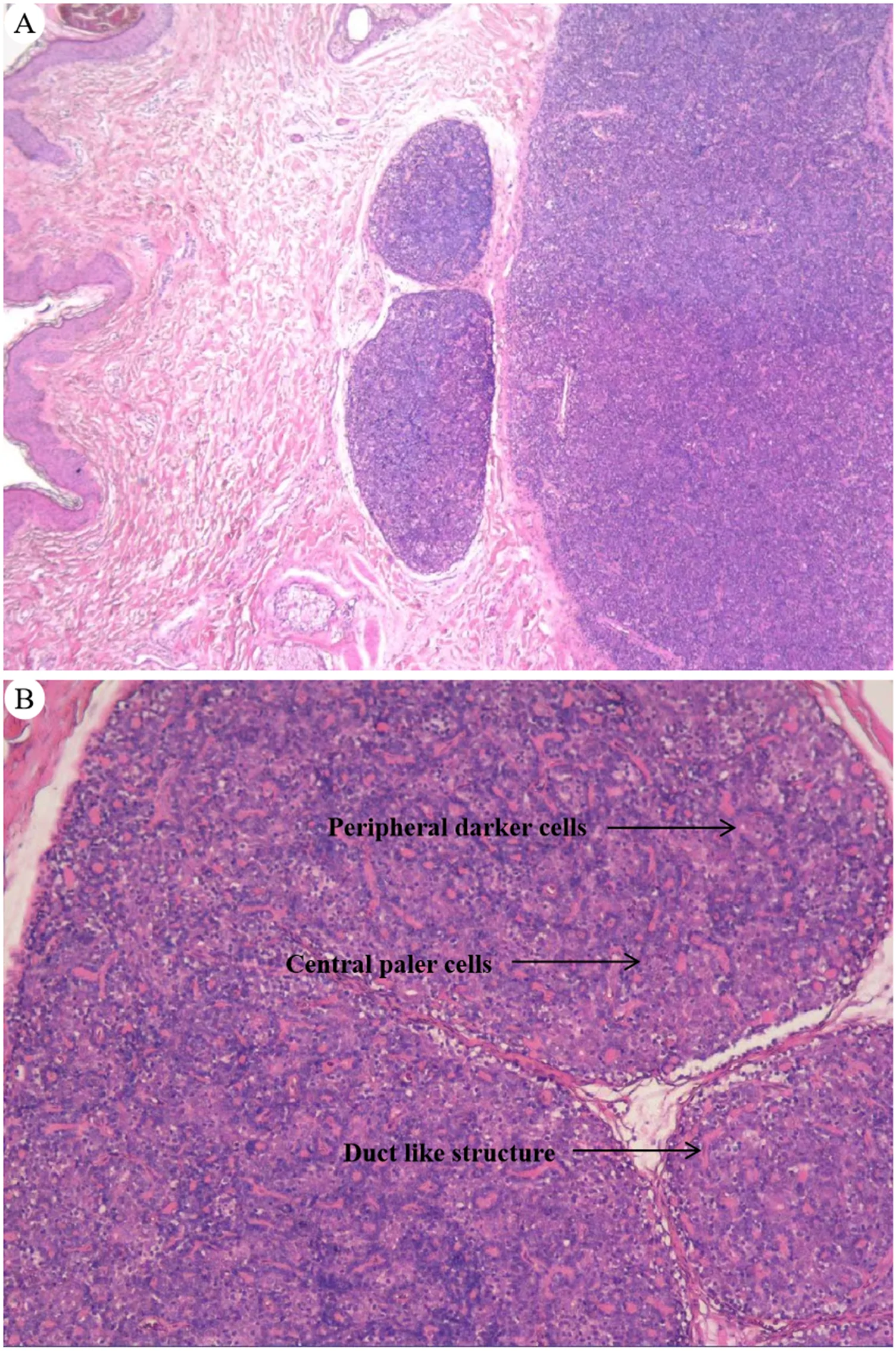
Histopathology. Hematoxylin and eosin images showing a lobulated dermal tumor with peripheral darker basaloid cells, central paler epithelial cells, and focal duct-like structures. **(A)** Original magnification ×40. **(B)** Original magnification ×200.

The postoperative course was uneventful, with primary wound healing and complete relief of local tenderness (Figure 1D). At the 12-month follow-up, no clinical recurrence or other local complication was observed (Figure 1E). The scar remained broad and visible. The Vancouver Scar Scale score was 4, with 1 point each for vascularity, pigmentation, height, and pliability. Continued clinical surveillance was advised because follow-up remains limited to 12 months. A brief timeline of care is shown in Table 1.

**Table 1:** Clinical timeline.

Date	Event
12 April 2024	Color Doppler ultrasonography suggested a vascular malformation with possible thrombosis.
15 May 2024	Contrast-enhanced CT was interpreted as a vascular lesion, reported as “subcutaneous plexiform hemangioma.”
16 May 2024	Complete excision and rhomboid local flap closure were performed.
17 May 2024	Histopathology confirmed eccrine spiradenoma.
12-month follow-up	No clinical recurrence was observed; the scar remained visible and continued follow-up was advised.

## Patient perspective

At follow-up, the patient reported complete relief of the preoperative tenderness and no new local symptoms. The scar remained visible, and no formal patient-reported cosmetic score was collected. He was advised to continue clinical follow-up.

## Discussion

The main diagnostic difficulty in this case was the vascular appearance on imaging. ES typically presents as a painful dermal or subcutaneous nodule (1, 2), and ultrasonography often demonstrates a solid hypoechoic mass with posterior enhancement and variable internal vascularity (3–6). A 2024 report of scapular vascular ES similarly showed that prominent vascular features can obscure the adnexal origin of a shoulder lesion (12).

In this patient, intralesional Doppler flow and a serpiginous, beaded CT pattern led to vascular interpretations at both institutions. Similar vascular ES may resemble angiomatous lesions or thrombosed veins (7–12). Current International Society for the Study of Vascular Anomalies terminology distinguishes vascular tumors from vascular malformations (13). We therefore retain “subcutaneous plexiform hemangioma” only as the wording of the original CT report and use the broader term “vascular lesion” when discussing the preoperative impression.

For a tender superficial mass, persistence of a solid component in the deep dermis or subcutaneous tissue should keep an adnexal tumor in the differential, even when Doppler flow or enhancement is conspicuous. Features such as compressibility, enlargement with dependency, slow flow, and phleboliths support a venous malformation, whereas a solid painful lesion with internal vascularity should prompt consideration of ES and other adnexal tumors. Yoshida et al. reviewed multiple vascular ES and emphasized that vascularity can occur within the ES spectrum (14). Figure 3 summarizes these clinical considerations for illustration only and is not a validated diagnostic algorithm.

**Figure 3:**
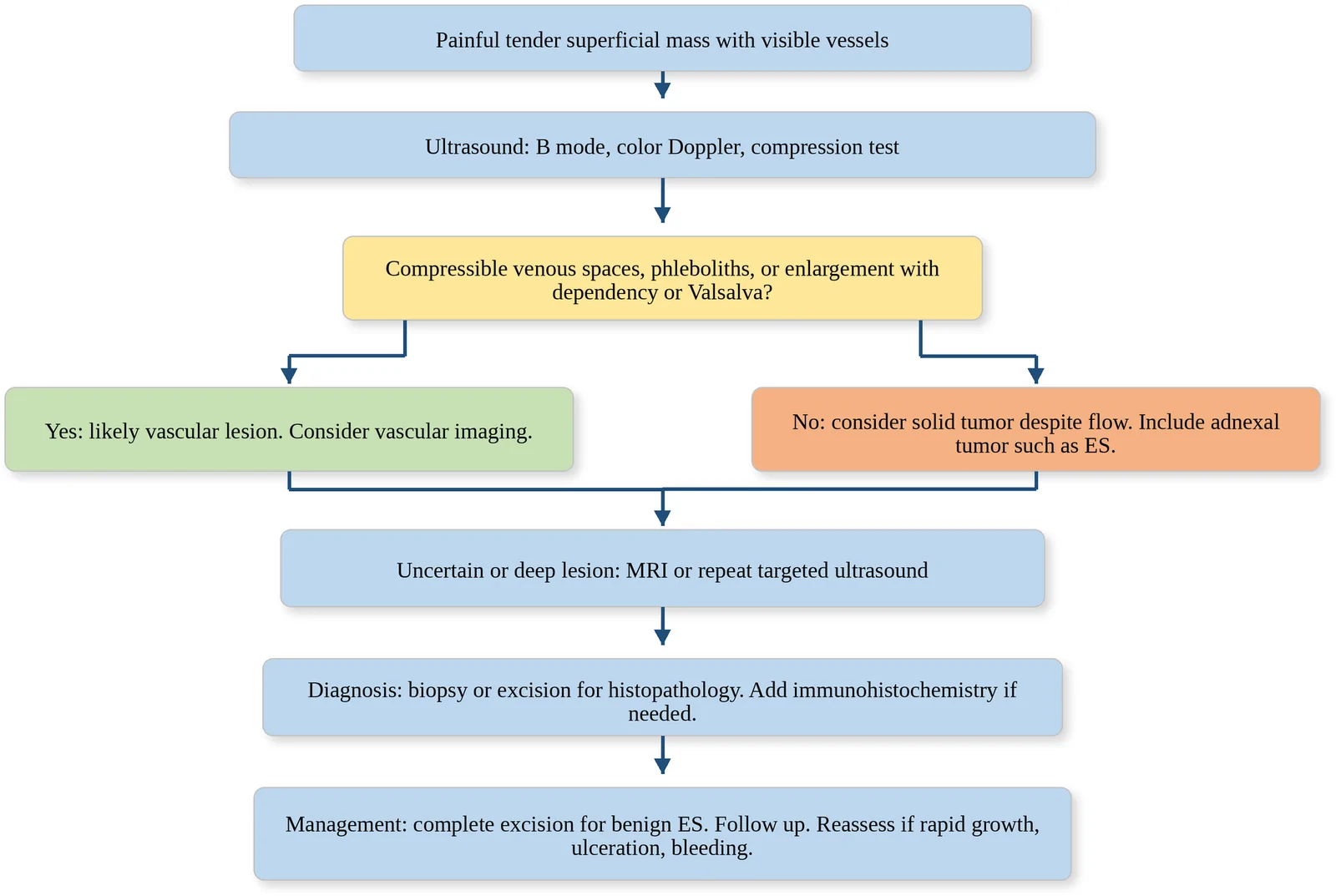
Illustrative clinical considerations for painful superficial masses with apparent vascularity (the figure summarizes considerations from this case and the cited literature and is not a validated diagnostic algorithm).

Histopathology remains central to diagnosis. Classic ES shows lobulated architecture with small darker basaloid cells at the periphery, larger paler cells centrally, and focal ductal differentiation. When morphology is equivocal, immunohistochemistry may support lineage and differentiation. Reported patterns include CK7 and epithelial membrane antigen in luminal epithelial cells, p63 in basal cells, and smooth muscle actin in myoepithelial cells. CK19 and carcinoembryonic antigen may highlight ductal or luminal differentiation, while S100 staining is variable (8, 9, 15). These markers are supportive rather than specific because overlap with cylindroma is recognized (15). Distinction from spiradenocarcinoma relies mainly on malignant morphologic features rather than a single immunostain (16–18). In this case, the hematoxylin and eosin morphology was considered diagnostic by the pathology team. Immunohistochemistry was not performed because the patient declined further testing.

Complete excision remains the usual treatment for benign ES (2). In this patient, the rhomboid flap was used to close the defect and reduce tension. At 12 months, the scar remained broad and visible, with a Vancouver Scar Scale score of 4. The reconstructed area remained accessible to inspection and palpation. No recurrence was observed, but this represents a short-term finding. Longer follow-up is warranted because rare malignant transformation has been reported. Rapid growth, ulceration, bleeding, recurrent pain, or a new mass should prompt reassessment (16–18).

This report has several limitations. No preoperative clinical photograph was retained. The archived ultrasound study did not preserve compression testing, quantitative Doppler indices, or representative spectral waveforms, and the CT report did not specify the contrast phase. Immunohistochemistry was not performed, and follow-up is limited to 12 months.

## Data Availability

The original contributions presented in the study are included in the article; further inquiries can be directed to the corresponding author.
